# Supplementary material for: Betaine: A Potential Nutritional Metabolite in the Poultry Industry
Source: Animals (Basel). 2022 Sep 30;12(19):2624. doi: 10.3390/ani12192624 (PMC9559486; doi:10.3390/ani12192624)
Supplement: Supplementary file 1 [file animals-12-02624-s001.zip › animals-1906515-supplementary.pdf]

animals

Review

Betaine: A Potential Nutritional Metabolite in the Poultry Industry

Wafaa A. Abd El-Ghany [1,†] and Daryoush Babazadeh [2,*,†,‡]

[1] Poultry Diseases Department, Faculty of Veterinary Medicine, Cairo University, Giza 12211, Egypt;
[2] Faculty of Veterinary Medicine, Shiraz University, Shiraz P.O. Box 71964-84334, Iran
* Correspondence: daryoushbabazadeh@shirazu.ac.ir; daryoushbabazadeh@gmail.com
† ORCID: 0000-0003-1686-3831 (W.A.A.E.-G.); ORCID: 0000-0003-1118-2295 (D.B.)
‡ Tel.: +98-9147697527

Citation: El-Ghany, W.A.A.; Babazadeh, D. Betaine: A Potential Nutritional Metabolite in the Poultry Industry. Animals 2022, 12, 2624. https://doi.org/10.3390/ani12192624

Academic Editor: Anna Arczewska-Włosek

Received: 24 August 2022
Accepted: 26 September 2022
Published: 30 September 2022

Publisher's Note: MDPI stays neutral with regard to jurisdictional claims in published maps and institu-tional affiliations.

Copyright: © 2022 by the authors. Submitted for possible open access publication under the terms and conditions of the Creative Commons Attribution (CC BY) license (https://creativecommons.org/licenses/by/4.0/).

Table S1. Effects of dietary supplementation of betaine on the growth parameters of broiler chickens.

Concentration of betaine/kg diet	Effect	Reference
0.05-0.15 mg/kg	Improvement of average body weight gain	73, 108
0.5-1 g/kg	Significant positive effects on body weight gain, feed intake, feed conversion rate, and viability rate	45, 94
1-2 g/kg	Significant improvement in the body weight, body weight gain, and feed conversion ratio and reduced mortality rate	19, 30, 61, 94, 110, 135, 142
2-4 g/kg	Significant positive effects on body weight gain	111
800 mg/kg + methionine 15, 18, 20, 22, and 24 g/kg	Significant increase in body weight gain at 21 days of age	51
In ovo injection at dosage of 0.25 ml with 0.5 ml deionized water	Improved hatching weight and final weight	109
